# Mitochondrial apoptosis-related gene polymorphisms are associated with responses to anthracycline-based chemotherapy in acute myeloid leukaemia

**DOI:** 10.3389/fonc.2023.1179937

**Published:** 2023-07-04

**Authors:** Guangqiang Meng, Mingying Li, Yuan Xia, Yuyan Wu, Yuechan Ma, Min Ji, Jingru Zhang, Jingjing Ye, Tao Sun, Chunyan Ji

**Affiliations:** ^1^ Department of Hematology, Qilu Hospital, Shandong University, Jinan, China; ^2^ Shandong Key Laboratory of Immunohematology, Qilu Hospital, Shandong University, Jinan, China

**Keywords:** acute myeloid leukemia, mitochondrial apoptosis, single nucleotide polymorphisms, anthracyclines, CRISPR/Cas9

## Abstract

**Background:**

Although anthracyclines are the first-line chemotherapy drugs for treating non-M3 acute myeloid leukaemia (AML), their efficacy remains limited. It is important to identify factors that influence the efficacy of anthracyclines against AML. Mitochondrial apoptosis-related genes play significant roles in the pathogenesis, treatment, and prognosis of AML.

**Methods:**

We utilized the CRISPR/Cas9 screening system to find AML anthracyclines resistance related genes and several mitochondrial apoptosis-related genes, such as *BCL2L11*, *CASP8*, *TP63*, *TP53BP2*, *PLAUR*, *SOD2*, *BNIP3L*, and *MMP9*, were screened out. Then, DNA from 279 patients with AML and 321 healthy individuals were extracted and the contributions of single nucleotide polymorphisms (SNPs) within these genes to the patient’s chemotherapy response, susceptibility to AML, and overall survival were investigated.

**Results:**

Our findings indicated that SNP rs4251864 in the *PLAUR* gene was associated with an increase in complete remission after anthracycline-based induction chemotherapy. rs4880 in *SOD2* was associated with the response to the second course of chemotherapy, whereas rs3789068 in *BCL2L11* was associated with susceptibility to AML.

**Conclusions:**

Our results about the association of SNPs in mitochondrial apoptosis-related genes with the response to anthracycline-based chemotherapy in AML provide an important reference for predicting the treatment outcomes in patients with this disease.

## Introduction

Acute myeloid leukaemia (AML), a heterogeneous myeloid malignancy, is characterised by the uncontrolled clonal proliferation of undifferentiated myeloid cells. Clinically, induction chemotherapy is needed as soon as possible to induce remission to save the life of patients with this disease. Although anthracycline- and cytarabine-based chemotherapy regimens are still the first-line treatments for non-M3 AML, the complete remission (CR) rates resulting from these drugs are only approximately 70–80% (1, 2). Primary resistance to chemotherapy drugs and relapse remain the main causes of death in patients with AML. Therefore, the search for strategies to further improve the CR rate has been pursued and discussed by clinicians. An important step towards this goal is to be able to evaluate the sensitivity of patients to induction chemotherapy in advance. For example, other new targeted therapies can be selected in advance for patients who are not sensitive to chemotherapy or have primary drug resistance. For this reason, we chose to target the anthracyclines, searching for indicators that could predict and assess a patient’s susceptibility to this class of drugs in advance.

To more accurately explore the bioindicators that affect the activity of anthracyclines against AML, we performed clustered regularly interspaced short palindromic repeats (CRISPR)/CRISPR-associated protein 9 (Cas9)-based screening for candidate genes after the exposure of THP-1 cells to the anthracycline drug idarubicin (IDA). Then, we used functional pathway enrichment analysis to identify candidate molecules related to the mitochondrial apoptosis pathway, which has been shown to be involved in the pathogenesis, treatment, and prognosis of AML (3, 4). The blocking or evasion of apoptosis in myeloid blasts is considered to be one of the characteristics of AML (4). For example, the anti-apoptotic protein B-cell lymphoma 2 (BCL2) is highly expressed in AML. Venetoclax, a BCL2 inhibitor, has shown good efficacy in the treatment of AML and has become the first-line treatment option for elderly patients with this disease (5–7). Furthermore, the tumour protein TP53, one of the molecules of the mitochondrial apoptosis pathway, is an important indicator of a poor treatment effect and poor survival prognosis in AML (8, 9). Therefore, mitochondrial pathway-related molecules are closely related to AML, and there may be molecules that predict the response or susceptibility to anthracycline-based chemotherapy.

AML is a heterogeneous disease. In addition to the differences in the pathogenesis of AML, its treatment is characterized by individual differences in sensitivity to chemotherapy drugs. As individual genetic variations, single nucleotide polymorphisms (SNPs) in AML patients are considered to be one of the important reasons for individual sensitivity to chemotherapy drugs (10, 11). Some genetic polymorphisms have been found to be associated with a patient’s susceptibility to AML and response to drugs, such as an increased risk of chemotherapy resistance (10–12). For example, SNPs of the P-glycoprotein-encoding *ABCB1* gene were found to predict the outcome of chemotherapy in AML patients (13, 14). Moreover, SNPs of genes coding for cytarabine metabolism enzymes are related to the treatment effect on patients with this disease (15). In this study, CRISPR-Cas9 screening and SNP sites analysis were employed to identify genes that may predict the response of AML cells to anthracycline chemotherapy and to seek the related SNP sites in these genes.

## Methods

### Patients and controls

In total, 279 patients with newly diagnosed AML (non-M3) were recruited from the Department of Hematology, Qilu Hospital of Shandong University from June 2011 to December 2021. All patients (median age: 49 years; range: 13–87 years) were diagnosed in accordance with the World Health Organization classification system and National Comprehensive Cancer Network (NCCN) guideline criteria (16, 17). The treatment response of AML was assessed according to the 2017 European Leukemia Network criteria (18). Overall survival (OS) was defined as the time from diagnosis to death or to the date of last follow-up in living patients censored. Additionally, 321 healthy individuals (median age: 40 years; range: 20–88 years) were enrolled as the control group. The characteristics of the AML patients and healthy individuals are shown in **Table 1**. This study was approved by the Ethics Committee of Qilu Hospital, Shandong University. Informed consent was obtained from all study participants in accordance with the Declaration of Helsinki.

**Table 1:** Demographic and clinical characteristics.

Variable	AML patients	Control
Sex (M/F)	159/120	120/201
Age (years, median, range)	49 (13-87)	40 (20-88)
FBA subtype (n, %)		
M0	1 (0.4)	N/A
M1	4 (1.4)	N/A
M2	22 (7.9)	N/A
M4	58 (20.8)	N/A
M5	186 (66.7)	N/A
M6	2 (0.7)	N/A
M7	0	N/A
Unknown	6 (2.2)	N/A
Cytogenetics and molecular stratifcation		
Low risk (n, %)	60 (21.5)	N/A
Intermediate risk (n, %)	145 (52.0)	N/A
High risk (n, %)	74 (26.5)	N/A
Response after the first course of induction therapy		
CR (n, %)	108 (56.8)	N/A
PR+NR (n, %)	82 (43.2)	N/A
Response after the second course of chemotherapy		
CR (n, %)	129 (80.1)	N/A
PR+NR (n, %)	32 (19.9)	N/A
Relapse after CR		
Yes (n, %)	40 (31.0)	N/A
No (n, %)	89 (69.0)	N/A

AML, acute myeloid leukemia; FAB, French–American–British classification; CR, complete remission; PR, partial remission; NR, no remission; N/A, not applicable.

### CRISPR/Cas9 screening

**Figure 1:**
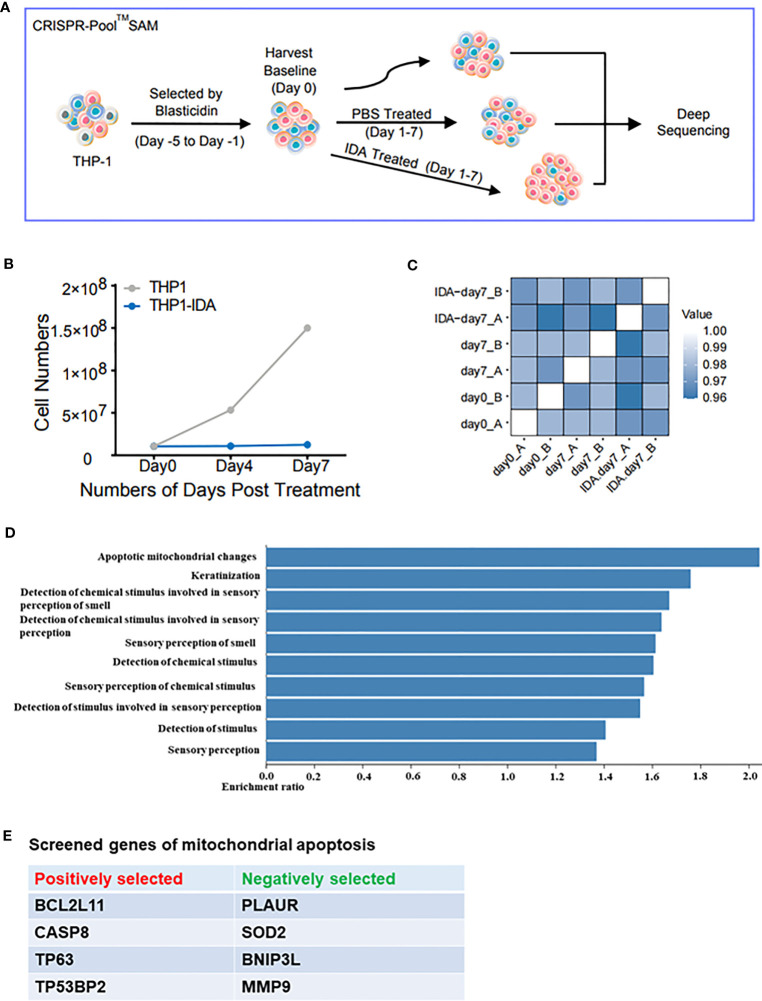
Mitochondrial apoptosis-related genes affect the killing of acute myeloid leukaemia cells by IDA. **(A)** The target genes of CRIPSR/Cas9 were screened in THP-1 cells. This screening process was based on the changes in sgRNA levels between the low-dose IDA group and untreated group. **(B)** Growth of THP-1 cells treated with IDA or PBS over 7 days. **(C)** Rank correlation of the normalised sgRNA read count between biological replicates and treatment conditions. **(D)** GO enrichment analysis of the screened genes showed that the proportion related to the mitochondrial apoptosis pathways was the highest. **(E)** Potential genes affecting the efficacy of IDA were selected from mitochondrial apoptosis-related genes. The genes with positive (resistance) and negative selection (sensitivity) were annotated.

For CRISPR/Cas9 screening with the CRISPR-Pool™ SAM vector (GeneChem, Shanghai, China), a CRISPR screening library was used, which contained 70,290 single guide RNAs (sgRNAs) targeting 23,430 genes and control genes, with an average of three sgRNAs per gene. THP-1 cells (a human AML monocytic cell line) were first transfected with the MS2-P65-HSF-Hygro lentivirus and then screened using 500 μg/mL hygromycin B (#V900372; Sigma-Aldrich, Beijing, China). Subsequently, these cells were infected with the sgRNA-dCas9-VP64-blasticidin lentivirus at a multiplicity of infection of approximately 0.25 and coverage rate of 300×, and the cells were cultured for 2 days. Then, the cells were treated with 4 μg/mL cysticercosin (#203351; Millipore, Beijing, China) for 72 h and cultured for 5 days to maintain a 300× coverage until the screening assay. After establishing day 0 as the baseline for sequencing, the THP-1 cells were divided into two groups: one group was grown in the absence of drug treatment as the untreated control, whereas the other group was grown in the continuous presence of low-dose IDA (3 ng/mL). The screening process lasted for 7 days. The two groups of cells were collected and used for high-throughput sgRNA sequencing, and the genes with large differences in sgRNA expression levels between the two cell groups were screened (**Figure 1**).

### DNA extraction and genotyping

A commercial DNA extraction kit (TianGen, Beijing, China) was used to extract genomic DNA from bone marrow-derived mononuclear cells. The concentration and purity of the extracted DNA were detected using a spectrophotometer. The SNPs of the selected genes are listed in **Table 2**. The genotypes of the selected SNPS were detected using a time-of-flight mass spectrometry (MassARRAY) system (BGI Technology, Shenzhen, China) and analyzed using multiplex polymerase chain reactions (PCRs), single-base extension reactions, resin purification, and mass spectrometry.

**Table 2:** Selected genes and SNPs.

Genes	SNPs
**BCL2L11**	rs724710
	rs3789068
**CASP8**	rs1045485^*^
**TP63**	rs4488809
	rs10937405
**TP53BP2**	rs10916264
	rs798755
	rs1538140
**PLAUR**	rs4251864
**SOD2**	rs5746136
	rs8031
**BNIP3L**	rs1055806^*^
**MMP9**	rs1056628^*^

SNP, single-nucleotide polymorphisms; *SNPs were not included in further analyses of Hardy-Weinberg equilibrium (HWE) deviations or were not suitable for the HapMap project.

### RNA extraction and real-time RT-PCR

Total RNA was extracted from mononuclear cells using the TRIzol reagent (Invitrogen, Carlsbad, CA, USA), and converted into cDNA using the PrimeScript RT Reagent Kit Perfect Real Time (Takara Bio, Japan). Quantitative PCR was performed on a LightCycler 480II Real-Time PCR system (Roche, Switzerland) following the standard protocol. At the end of the reactions, the cycle threshold (CT) was measured and the mean CT of three independent replicates was calculated. GAPDH was used as a reference gene.

### Statistical analysis

For all SNPs, tests for deviation from Hardy–Weinberg equilibrium (HWE) were performed using Pearson’s goodness-of-fit chi-square test. SNPs with an HWE *P-*value of greater than 0.05 and a minor allele frequency of greater than 5% were included in the study. To determine the relationships of the SNP phenotype or allele frequency with the susceptibility to AML, risk stratification, CR, relapse, and OS, the following statistical methods were used: the chi-square test or Fisher’s exact test for preliminary screening; univariate and multivariate binary logistic regression analyses for determining the odds ratios (ORs) with 95% confidence intervals (95%CI) and *P*-values adjusted for sex and age; Kaplan–Meier curves for estimating OS; and Cox regression analysis for evaluating the prognostic factors of AML. SPSS 20.0 software (SPSS Inc., Chicago, IL, USA) was used for all statistical analyses. Statistical significance was considered on the basis of either a two-tailed *P*-value of less than 0.05 or a false discovery rate (FDR) *q*-value of less than 0.05.

## Results

### Genes and single nucleotide polymorphisms

The THP-1 cells that had been treated with IDA for 7 days and the untreated control cells were harvested for high-throughput sgRNA sequencing. Genes with highly variable sgRNA expression levels between the two cell groups were considered to potentially contribute to IDA resistance. Firstly, we conducted functional pathway enrichment analysis of the differentially expressed genes and found that most were concentrated in the mitochondrial apoptosis-related pathways. Then, eight of the most promising genes in these pathways were screened out (**Figure 1**). The analysis of the sequencing results was a collaborative effort between the bioinformatics technicians and the research team. Combined with the results of previous studies, we further screened a total of 13 SNPs in eight related genes for analysis (**Table 2**).

**Figure 2:**
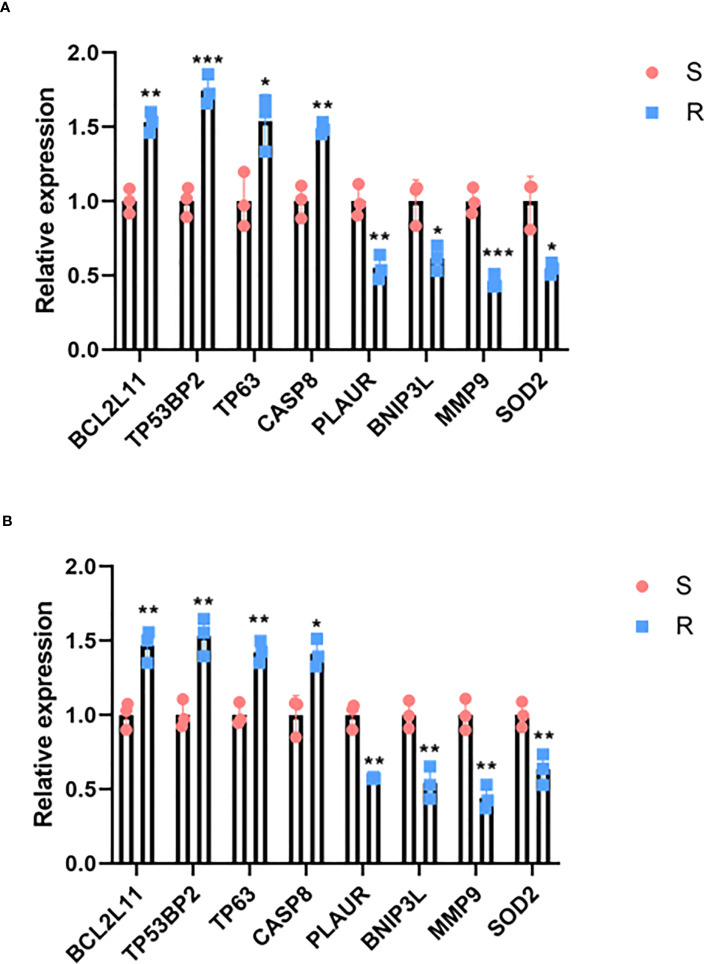
The mRNA expression levels of identified genes in sensitive and resistant cell lines. **(A)** The mRNA expression levels of identified genes in THP-1 sensitive (S) and resistant (R) cell lines. **(B)** The mRNA expression levels of identified genes in Kasumi-1 sensitive and resistant cell lines. *P < 0.05, **P < 0.01,***P < 0.001.

In order to confirm the effect of the selected genes on sensitivity to IDA, RT-PCR was performed to determine the expression of screened genes in THP-1 and Kasumi-1 cell lines that developed resistance to IDA. These two IDA-resistant AML cell lines were induced and established by our research group (19). The expression levels of the four resistant genes (*BCL2L11*, *CASP8*, *TP63*, and *TP53BP2)* screened by CRISPR/Cas9 were increased in both resistant cell lines, while the expression levels of the four sensitive genes (*PLAUR*, *SOD2*, *BNIP3L*, and *MMP9)* were decreased in resistant cell lines (**Figure 2**). This result was consistent with the results of CRISPR/Cas9 screening.

### Study population

The clinical and demographic characteristics of the AML patients and healthy participants are shown in **Table 1**. The SNPs rs1045485 in *CASP8*, rs1055806 in *BNIP3L*, and rs1056628 in *MMP9* were not included in further analyses for HWE deviations or were not suitable for the HapMap project (**Table 2**).

### Polymorphisms associated with response to anthracycline-based induction chemotherapy in acute myeloid leukaemia

The standard induction chemotherapy regimen for AML containing anthracycline was defined as anthracycly-based chemotherapy regimen (16, 17). The main anthracyclines used in chemotherapy for our AML patients were daunorubicin, idarubicin, and aclarubicin. The anthracycline-based chemotherapy regiments were DA, IA, CAG and HAA. A total of 190 (68.1%) out of 279 AML patients received anthracycline-based induction chemotherapy. Among them, 45, 104, 23 and 18 AML patients selected DA, IA, CAG and HAA induction chemotherapy regimen, respectively (**Table 3**). After the first course of treatment, 108 (56.84%, 108/190) patients achieved CR. To evaluate the association between the SNPs and response to the first course of induction chemotherapy, 190 patients were divided into CR and non-CR groups based on their response to the drug. A preliminary screening analysis using the chi-square or Fisher’s exact test revealed a significant association between rs4251864 in the plasminogen activator and urokinase receptor (*PLAUR*) gene and susceptibility to AML in both co-dominant and recessive models (*P =* 0.003 and *P =* 0.007, respectively). Univariate logistic regression analysis also confirmed the association between rs4251864 and susceptibility to AML in the co-dominant model (*P =* 0.015). After adjusting for sex and age and FDR correction, the recessive *GA* genotype of rs4251864 was found to be significantly associated with susceptibility to AML, with an increase in CR (*P* = 0.015) (**Table 4**).

**Table 3:** Anthracycline-based induction chemotherapy regimens and treatment response.

Regimen	Cases (n)	CR (n)
DA	45	24
IA	104	62
CAG	23	15
HAA	18	7

DA, daunorubicin-cytarabine; IA, idarubicin-cytarabine; CAG, cytarabine-aclarubicin-granulocyte colony-stimulating factor; HAA, Homoharringtonine-cytarabine-aclarubicin; CR, complete remission.

**Table 4:** Association between response and SNPs after first course of anthracycline-based induction chemotherapy in AML.

Gene	SNPs	Model	Genotype/allele	CR (n)	PR+NR (n)	OR (95% CI)	Adjusted *P*-value
**PLAUR**	rs4251864	Co-dominant	AA	65	55		
			GA	39	16	0.234 (0.069-0.796)	0.015
			GG	4	12	1.233 (0.649-2.342)	0.522
		Recessive	AA/GA	104	70		
			GG	4	12	0.248 (0.076-0.811)	0.051

SNP, single nucleotide polymorphisms; AML, acute myeloid leukemia; CR, complete remission; PR, partial remission; NR, no remission; CI, confidence interval; OR, odds ratio.

### Polymorphisms associated with the sensitivity to two courses of anthracycline-based chemotherapy in acute myeloid leukaemia

Patients who failed to achieve complete remission after two courses of standard-dose chemotherapy were classified as having refractory AML, which usually has a poor prognosis and short survival time. Patients who received anthracycline-based chemotherapy (180) were divided into the CR and non-CR groups. After the first course of chemotherapy, 161 patients received a second chemotherapy course, whereas the remaining 19 patients either refused chemotherapy or succumbed to the disease. At the end of the second chemotherapy course, 129 patients achieved CR, whereas the remaining 32 patients did not. Preliminary screening with the chi-square test or Fisher’s exact test revealed a significant correlation between the rs8031 SNP in the superoxide dismutase 2 (*SOD2*) gene and susceptibility to refractory AML under the co-dominant and recessive models (*P =* 0.006 and *P =* 0.006, respectively). Univariate logistic regression analysis also indicated an association between rs8031 and susceptibility to refractory AML in both the models (*P* = 0.015 and *P* = 0.011, respectively). The co-dominant *AT* and recessive *TT* genotypes of rs8031 were significantly associated with susceptibility to refractory AML after adjusting for sex and age (*P* = 0.015 and *P* = 0.011, respectively). The results were statistically significant after FDR correction (**Table 5**).

**Table 5:** Association between response and SNPs after second chemotherapy in AML.

Gene	SNPs	Model	Genotype/allele	CR (n)	PR+NR (n)	OR (95% CI)	Adjusted *P*-value
**SOD2**	rs8031	Co-dominant	AA	94	23		
			AT	34	5	16.174 (1.725-151.674)	0.015
			TT	1	4	0.578 (0.204-1.638)	0.302
		Recessive	AA/AT	128	28		
			TT	1	4	18.266 (1.968-169.907)	0.011

SNP, single nucleotide polymorphisms; AML, acute myeloid leukemia; CR, complete remission; PR, partial remission; NR, no remission; CI, confidence interval; OR, odds ratio.

### Polymorphisms associated with susceptibility to acute myeloid leukaemia

We analyzed the associations between the selected SNPs and susceptibility to AML using four genetic models (co-dominant, dominant, recessive, and allele), where the relationship between each locus and AML was analyzed. Preliminary screening with the chi-square test or Fisher’s exact test showed that the SNPs rs3789068 in the Bcl-2-like protein 11 (*BCL2L11*) gene (co-dominant, dominant, and allele models), rs724710 in *BCL2L11* (allele model), rs798755 in the tumour protein P53 binding protein 2 (*TP53BP2*) gene (co-dominant model), and rs4251864 in *PLAUR* (dominant and allele models) were significantly correlated with susceptibility to AML (*P* < 0.05). Univariate logistic regression analysis showed associations of the following SNPs with susceptibility to AML: rs3789068 in *BCL2L11* under the co-dominant, dominant, and allele models (*P* < 0.05); rs724710 in *BCL2L11* under the allele model (*P* = 0.027); and rs4251864 in *PLAUR* under the dominant and allele models (*P* = 0.045 and *P* = 0.043, respectively). All afore-mentioned univariate logistic regression analyses were adjusted for sex and age. However, after FDR correction, the co-dominant *GG* genotype and *G* alleles of rs3789068 in *BCL2L11* were found to be associated with susceptibility to AML (*q* < 0.05) (**Table 6**).

**Table 6:** Association between selected SNPs and susceptibility of AML.

Gene	SNPs	Model	Genotype/allele	Cases (n)	Controls (n)	OR (95% CI)	Adjusted *P*-value
BCL2L11	rs3789068	Co-dominant	AA	76	123		
			GA	144	145	1.53 (0.948-2.470)	0.082
			GG	59	53	1.655 (1.129-2.428)	0.010
		Dominant	AA	76	123		
			GA/GG	203	198	1.654 (1.153-2.370)	0.006
		Allele	A	296	391		
			G	262	251	1.348 (1.061-1.713)	0.014
	rs724710	Allele	C	497	548		
			T	60	94	0.64 9 (0.455-0.926)	0.027
TP53BP2	rs798755	Co-dominant	GG	231	250		
			GA	42	69	3.658 (0.718-18.639)	0.118
			AA	6	2	0.744 (0.479-1.156)	0.189
PLAUR	rs4251864	Dominant	AA	155	150		
			GA/GG	124	171	0.710 (0.508-0.993)	0.045
		Allele	A	415	440		
			G	143	202	0.763 (0.586-0.992)	0.043

SNP, single nucleotide polymorphisms; AML, acute myeloid leukemia; CI, confidence interval; OR, odds ratio.

### Polymorphisms associated with risk stratification of acute myeloid leukaemia

Risk stratification based on molecular, genetic, and cytogenetic changes plays a significant role in guiding the prognosis of AML. Patients with AML were divided into groups with favourable, intermediate, or adverse prognosis based on karyotypic and molecular abnormalities based on NCCN guidelines. We detected no significant association of the selected SNPs with prognostic stratification after initial screening using the chi-square test or Fisher’s exact test (*P* > 0.05) (**Supplementary Table 1**).

### Polymorphisms associated with sensitivity to acute myeloid leukaemia relapse

After two courses of anthracycline-based chemotherapy, 129 patients achieved CR, but 40 of them relapsed during treatment or follow-up. We also analyzed the association of the selected SNPS with sensitivity to AML relapse using the chi-square test or Fisher’s exact test but found no such association with any of them (*P* > 0.05) (**Supplementary Table 2**).

### Associations of single nucleotide polymorphisms with survival in acute myeloid leukaemia

**Figure 3:**
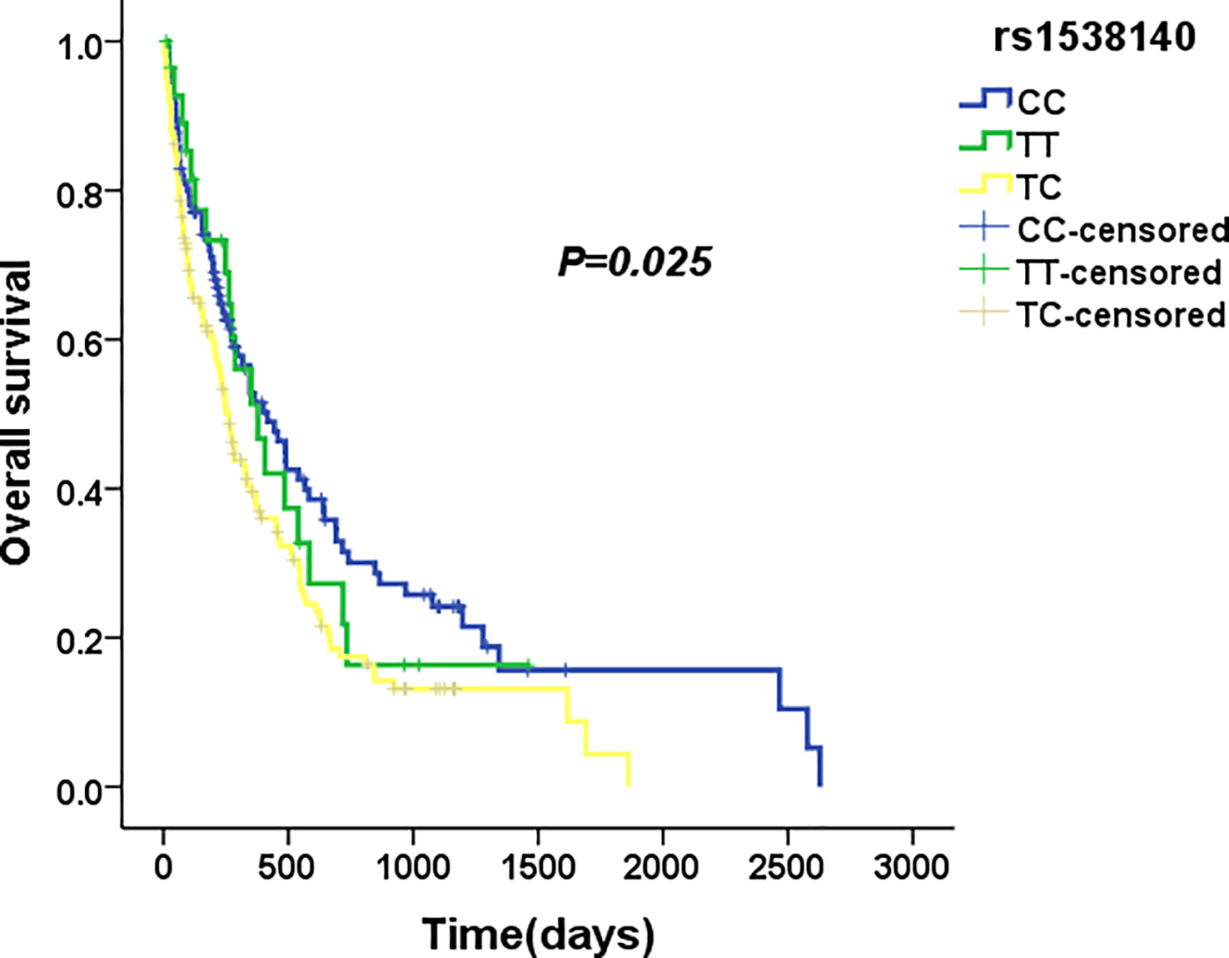
The median overall survival of AML patients with the CC, TC and TT genotypes in rs1538140.

**Figure 4:**
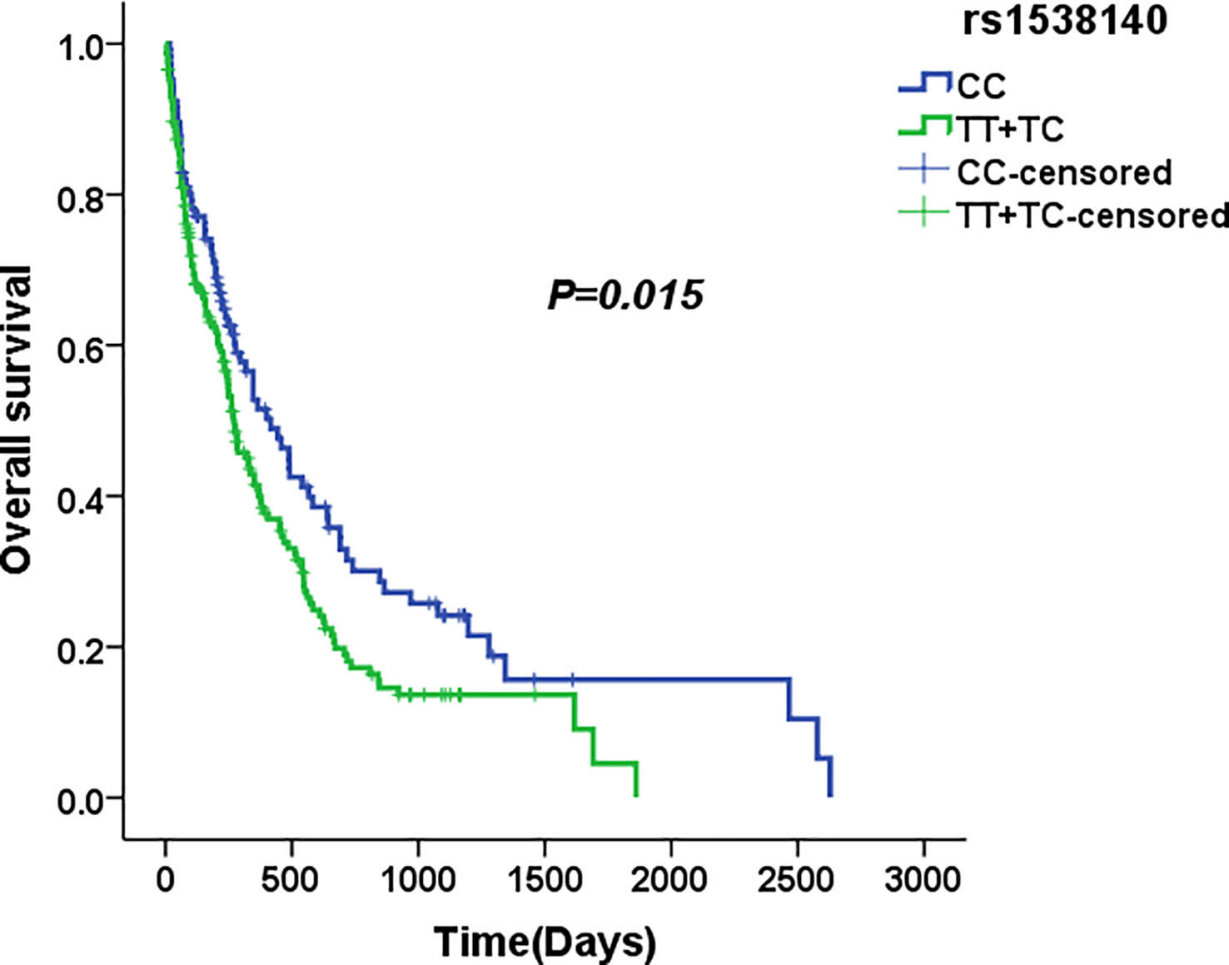
The median overall survival of AML patients with the CC and TC+TT genotypes in rs1538140.

We used four genetic models to analyse the association between various SNPs and survival of AML patients. Preliminary Kaplan–Meier screening revealed an association between the genotype frequency of rs1538140 in *TP53BP2* and disease prognosis in the dominant and co-dominant models (*P* = 0.025 and *P* = 0.015, respectively) (**Figures 3**, **4**). The other SNPs had no significant effect on survival. Patients with the *CC* genotype had prolonged OS compared with patients with the *TT* and *TC* genotypes under the co-dominant model of rs1538140 in *TP53BP2*. Furthermore, the survival of patients with the *CC* genotype was significantly prolonged compared to that of patients with the *TT*/*TC* genotypes under the dominant model of rs1538140 in *TP53BP2*.

Patients aged 60 years or older had significantly lower OS (median = 198 days) than those in the younger age group (median = 380 days, *P* < 0.001). Additionally, patients with a white blood cell count (WBC) count higher than 100 × 10^9^ had significantly lower OS (median = 85 days) than those with a WBC count lower than 100 × 10^9^ (median = 416 days, *P* < 0.001). Patients who did not receive allogeneic haematopoietic stem cell transplantation (allo-HSCT) had significantly lower OS (mean = 538 ± 49.716 days) than those who received treatment (mean = 1046 ± 87.698 days, *P* < 0.001). Similarly, patients who did not receive chemotherapy also had significantly lower OS (median = 56 days) than those who received treatment (median = 400 days, *P* < 0.001). Additionally, OS was significantly lower in patients with adverse risk stratification (median = 213 days) than in those with favourable stratification (median = 657 days, *P* < 0.001).

The existence of rs1538140 SNP in *TP53BP2*, age, WBC count greater than 100 × 10^9^, risk stratification, chemotherapy option, and allo-HSCT option were included in multivariate regression analysis using the Cox regression analysis. The results of this analysis showed that the rs1538140 genotype is not an independent prognostic risk factor for AML. Moreover, adverse risk stratification (OR = 1.543, 95% CI = 1.245–1.914), age greater than 60 years (OR = 1.385, 95% CI = 1.006–1.906), WBC count higher than 100 × 10^9^/L (OR = 0.856, 95% CI = 0.786–0.932), no chemotherapy (OR = 0.221, 95% CI = 0.152–0.324), and no allo-HSCT (OR = 0.127, 95% CI = 0.031–0.513) were independent prognostic risk factors for AML (**Table 7**).

**Table 7:** Multivariate COX regression analysis of risk factors for overall survival of AML patients.

Risk factor	OR	95% CI	*P*-value
age greater than 60 years	1.385	1.006-1.906	0.046
WBC count higher than 100 × 10^9^/L	0.856	0.786-0.932	0.000
no allo-HSCT	0.127	0.031-0.513	0.004
no chemotherapy	0.221	0.152-0.324	0.000
adverse risk stratification	1.543	1.245-1.914	0.000

AML, acute myeloid leukemia; WBC, white blood cell; allo-HSCT, allogeneic hematopoietic stem cell transplantation; CI, confidence interval; OR, odds ratio.

### PLAUR and SOD2 mRNA expression in AML patients with CR

**Figure 5:**
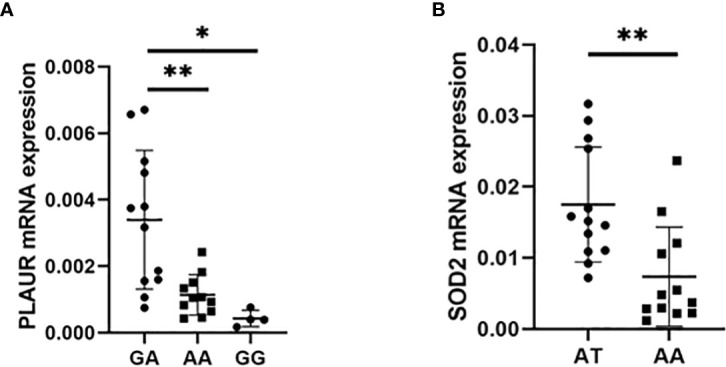
PLAUR and SOD2 mRNA expression in AML patients with CR. **(A)** Expression of PLAUR mRNA in AML patients with the GA, AA and GG genotype (n = 12, n = 11 and n = 4, respectively). **(B)** Expression of SOD2 mRNA in AML patients with the AT and AA genotype (n = 13 and n = 12, respectively). *P < 0.05, **P < 0.01.

To validate the association between the GA genotype of PLAUR rs4251864 and the response to the first induction chemotherapy containing anthracyclines, we selected 27 patients who achieved CR after the first induction chemotherapy, including 12 GA genotype, 11 AA genotype and 4 GG genotype. The mRNA expression level of PLAUR in bone marrow mononuclear cells was detected by RT-PCR. The results showed that the mRNA expression of PLAUR in AML patients with GA genotype was higher than that of patients with AA and GG genotype (P = 0.0023 and P = 0.0149) (**Figure 5A**). The finding confirmed that the GA genotype of rs4251864 is associated with a higher expression of PLAUR who would be responsible for the CR rate after being treated with the first course of induction chemotherapy. Meanwhile, we selected 25 patients who achieved CR after 2 courses of chemotherapy, including 13 AT genotype and 12 AA genotype of SOD2 rs4880. The SOD2 mRNA expression level in patients with AT genotype was higher than that in patients with AA genotype (P = 0.0028) (**Figure 5B**). Because there was only one TT genotype in CR patients after 2 courses of chemotherapy, statistical analysis could not be conducted. The results confirmed that the AT genotype of SOD2 rs8031 is associated with a higher expression of SOD2 who would be responsible for the higher CR rate after the second course of induction chemotherapy.

## Discussion

In this study, we investigated the association of 13 SNPs across eight genes involved in the mitochondrial apoptosis pathway with AML. Of these, 10 effective SNPs were further analysed in patients with AML and in healthy participants. A comprehensive statistical analysis revealed an association between the rs4251864 SNP in *PLAUR* and a decreased CR rate after anthracycline-based induction chemotherapy. The rs4880 SNP in *SOD2* was also found to be associated with the response to two courses of chemotherapy. The rs3789068 in *BCL2L11* was found to be associated with susceptibility of patients to AML. However, no association was found between the selected SNPs and AML risk stratification or disease recurrence. Patients with the *CC* genotype of the rs1538140 SNP in *TP53BP2* had better OS. However, the selected SNPs were not associated with patient survival in multivariate analysis. These results suggest an involvement of mitochondrial apoptosis-related genes in AML pathogenesis and development. Our findings thus may serve as potential therapeutic response indicators for guiding clinical treatment of this disease.

The influence of gene polymorphisms on the response to induction chemotherapy and targeted therapy in AML patients has been extensively studied. For example, researchers have found that SNPs in the *ABCB1* gene are significantly related to CR after chemotherapy, with some studies showing them to be related to an increase in the CR rate and others reporting the opposite effect (13, 14, 20). Moreover, SNPs in *ABCB1* were found to predict the outcome of gemtuzumab ozogamicin treatment in AML patients (21). In our study, rs4251864 in the *PLAUR* gene was associated with the response to the first course of anthracycline-based induction chemotherapy, with the co-dominant model suggesting the *GA* genotype of this SNP to be associated with an increase in the CR rate after chemotherapy. *PLAUR*, a membrane protein also known as urokinase-type plasminogen activator receptor (UPA-R, CD87), is mainly responsible for the expression of cellular plasmin, which promotes cell extravasation and tissue invasion. Studies have found that *PLAUR* is closely related to the occurrence and prognosis of a variety of malignant tumours (22, 23). Furthermore, *PLAUR* gene polymorphism also affects the prognosis of tumours, with SNPs in the gene found to be associated with disease-free survival in breast cancer (24). *PLAUR* has been reported to be highly expressed in AML and is associated with a poor prognosis (25). However, no studies have reported the correlation between *PLAUR* SNPs and AML. We used the CRISPR/Cas9 system to screen *PLAUR* for sensitivity to anthracyclines in an AML cell line. Additionally, this is the first report of *PLAUR* SNPs being associated with the response to the first course of anthracycline-based induction chemotherapy in AML, which has important implications for the use of this class of antibiotics in the treatment of this disease. However, the results still need to be further confirmed in studies with larger sample sizes.

Additionally, we found an association between a *SOD2* SNP and the response to the second course of chemotherapy. SOD2 is an antioxidative enzyme which plays an important role in the balance between oxidation and antioxidants in the body (26). Although the loss of a cell’s antioxidative capacity and the resulting intracellular oxidation-dependent damage are keys causes of tumourigenesis, effective oxidative clearance is necessary for tumour metastasis and spread (27). Therefore, SOD2 has dichotomous anti-tumourous and tumourigenesis effects in cancer. Although *SOD2* gene polymorphisms have been reported to be closely related to the susceptibility and prognosis of solid tumours, their effect on haematological tumours is rarely reported (28, 29). One study on acute lymphoblastic leukaemia reported an association between the *SOD2* gene variant rs4880 and hepatotoxicity in patients undergoing asparaginase-based therapy (29). However, the correlation between rs4880 and the treatment response in AML has not been reported. In our study, under the co-dominant model, the *AT* genotype of rs4880 was associated with increased CR after the second course of chemotherapy. However, under the recessive model, the *TT* genotype of this SNP was associated with increased no CR. The reasons may be as follows: (1) SOD2 itself has a dichotomous effect of both promoting and inhibiting the tumour; and (2) only a small number of patients did not respond to the second course of chemotherapy. Therefore, the influence of the *SOD2* gene polymorphism on the response to AML chemotherapy still needs to be confirmed in a study with a larger sample size.

Although many studies on the relationship between SNPs and AML prognosis have been carried out, there are few reports on that between SNPs and susceptibility to AML. In our study, the co-dominant *GG* genotype and dominant *GA*/*GG* genotypes of rs3789068 in *BCL2L11* were found to be significantly associated with susceptibility to AML. As a member of the *BCL2* family, *BCL2L11* plays a key role in regulating apoptosis in B- and T-cell homeostasis (30, 31). *BCL2L11* can bind to anti-apoptotic Bcl-2-like proteins and plays an important role in the initiation of apoptosis (32). However, it mainly plays a pro-apoptotic role, maintaining the homeostasis of haematopoietic cells by initiating the apoptosis of lymphocytes and balancing the proliferation and anti-apoptotic effects of *BCL2*. Therefore, single nucleotide mutations in *BCL2L11* may break the homeostasis of haematopoietic cells and lead to haematological malignancy. The role of rs3789068 in inducing susceptibility to haematological malignancies has been reported. In a study involving 5330 patients with B-cell non-Hodgkin’s lymphoma and 6260 healthy individuals, rs3789068 was found to be associated with an increased risk of the disease (33). In another study, the rs3789068 mutation was found to be closely related to the development of follicular lymphoma (34). These studies are consistent with our finding of the association of this *BCL2L11* gene SNP with susceptibility to AML.

In this study, we innovatively combined CRISPR/Cas9 screening and SNP analysis to study the correlation between SNP sites and AML chemotherapy response, which greatly improves the reliability of the results and will be the biggest advantage of our study. Meanwhile, the molecular and cellular mechanisms of the identified genes that influence drug sensitivity worth for further study. In addition, owing to the heterogeneity of the AML patient population and the small number of patients in this study, the results should be further confirmed by studies with larger sample sizes.

## Conclusion

In this study, we proved that rs4251864 in PLAUR or rs4880 in SOD2 were associated with the response to first-induction chemotherapy or second-course chemotherapy of AML containing anthracyclines, respectively. The results suggested that the mitochondrial apoptosis-related pathways represented by PLAUR and SOD2 are very important for anthracycline sensitivity. Their SNPs can be used as predictors for the response to chemotherapy in AML patients. And we plan to further investigate the molecular and cellular mechanisms of the identified genes that influence drug sensitivity in future studies.

## Data availability statement

The data presented in the study are deposited in the GEO repository, accession number GSE235688.

## Ethics statement

This study was conducted in accordance with the Declaration of Helsinki and approved by the Medical Ethics Committee of Qilu Hospital, Shandong University. We obtained informed consent from the patients and controls involved in the study.

## Author contributions

CJ, TS, and GM designed the study; GM performed the research, analyzed the data, and drafted the manuscript. ML, YX, YW, YM, MJ, JZ, and JY performed the research and evaluated the data. All authors contributed to the article and approved the submitted version.
