# Supplementary material for: Mitochondrial apoptosis-related gene polymorphisms are associated with responses to anthracycline-based chemotherapy in acute myeloid leukaemia
Source: Front Oncol. 2023 Jul 4;13:1179937. doi: 10.3389/fonc.2023.1179937 (PMC10352653; doi:10.3389/fonc.2023.1179937)
Supplement: Supplementary file 1 [file DataSheet_1.docx]

**Supplementary** **table 1. Polymorphisms associated with risk stratification of acute myeloid leukaemia**

| Gene | SNPs | Model | Genotype/allele | Favourable | Intermediate | Adverse | chi-square test  P-value |
| --- | --- | --- | --- | --- | --- | --- | --- |
| **BCL2L11** | rs724710 | Co-dominant | CC | 48 | 113 | 58 |  |
|  |  |  | CT | 12 | 31 | 16 |  |
|  |  |  | TT | 0 | 1 | 0 | 0.98 |
|  |  | Dominant | CC | 48 | 113 | 58 |  |
|  |  |  | CT/TT | 12 | 32 | 16 | 0.962 |
|  |  | Recessive | CC/CT | 60 | 144 | 74 |  |
|  |  |  | TT | 0 | 1 | 0 | 1.000 |
|  |  | Allele | C | 108 | 257 | 132 |  |
|  |  |  | T | 12 | 33 | 16 | 0.904 |
|  | rs3789068 | Co-dominant | AA | 18 | 41 | 19 |  |
|  |  |  | GA | 30 | 71 | 41 |  |
|  |  |  | GG | 12 | 33 | 14 | 0.908 |
|  |  | Dominant | AA | 18 | 41 | 19 |  |
|  |  |  | GA/GG | 42 | 104 | 55 | 0.864 |
|  |  | Recessive | AA/GA | 48 | 112 | 60 |  |
|  |  |  | GG | 12 | 33 | 17 | 0.910 |
|  |  | Allele | A | 66 | 153 | 79 |  |
|  |  |  | G | 54 | 137 | 69 | 0.918 |
| **TP63** | rs4488809 | Co-dominant | TT | 16 | 46 | 19 |  |
|  |  |  | CT | 27 | 61 | 31 |  |
|  |  |  | CC | 17 | 38 | 24 | 0.821 |
|  |  | Dominant | TT | 16 | 46 | 19 |  |
|  |  |  | CT/CC | 44 | 99 | 55 | 0.619 |
|  |  | Recessive | TT/CT | 43 | 107 | 50 |  |
|  |  |  | CC | 17 | 38 | 24 | 0.647 |
|  |  | Allele | T | 59 | 153 | 69 |  |
|  |  |  | C | 61 | 137 | 79 | 0.456 |
|  | rs10937405 | Co-dominant | CC | 33 | 75 | 37 |  |
|  |  |  | TC | 20 | 54 | 29 |  |
|  |  |  | TT | 7 | 16 | 8 | 0.975 |
|  |  | Dominant | CC | 33 | 75 | 37 |  |
|  |  |  | TC/TT | 27 | 70 | 37 | 0.854 |
|  |  | Recessive | CC/TC | 53 | 129 | 66 |  |
|  |  |  | TT | 7 | 16 | 8 | 1.000 |
|  |  | Allele | C | 86 | 204 | 103 |  |
|  |  |  | T | 34 | 86 | 45 | 0.931 |
| **TP53BP2** | rs10916264 | Co-dominant | AA | 31 | 85 | 43 |  |
|  |  |  | GA | 25 | 50 | 29 |  |
|  |  |  | GG | 4 | 10 | 2 | 0.610 |
|  |  | Dominant | AA | 31 | 85 | 43 |  |
|  |  |  | GA/GG | 29 | 60 | 31 | 0.634 |
|  |  | Recessive | AA/GA | 56 | 135 | 72 |  |
|  |  |  | GG | 4 | 10 | 2 | 0.457 |
|  |  | Allele | A | 87 | 220 | 115 |  |
|  |  |  | G | 33 | 70 | 33 | 0.611 |
|  | rs798755 | Co-dominant | GG | 52 | 122 | 56 |  |
|  |  |  | GA | 8 | 19 | 15 |  |
|  |  |  | AA | 0 | 4 | 3 | 0.331 |
|  |  | Dominant | GG | 52 | 122 | 56 |  |
|  |  |  | GA/AA | 8 | 23 | 18 | 0.165 |
|  |  | Recessive | GG/GA | 60 | 141 | 71 |  |
|  |  |  | AA | 0 | 4 | 3 | 0.359 |
|  |  | Allele | G | 112 | 263 | 127 |  |
|  |  |  | A | 8 | 27 | 21 | 0.102 |
|  | rs1538140 | Co-dominant | CC | 26 | 55 | 24 |  |
|  |  |  | TC | 30 | 76 | 38 |  |
|  |  |  | TT | 4 | 14 | 12 | 0.376 |
|  |  | Dominant | CC | 26 | 55 | 24 |  |
|  |  |  | TC/TT | 34 | 90 | 60 | 0.163 |
|  |  | Recessive | CC/TC | 56 | 131 | 62 |  |
|  |  |  | TT | 4 | 14 | 12 | 0.188 |
|  |  | Allele | C | 82 | 186 | 86 |  |
|  |  |  | T | 38 | 104 | 62 | 0.215 |
| **PLAUR** | rs4251864 | Co-dominant | AA | 34 | 81 | 40 |  |
|  |  |  | GA | 22 | 52 | 31 |  |
|  |  |  | GG | 4 | 12 | 3 | 0.794 |
|  |  | Dominant | AA | 34 | 81 | 40 |  |
|  |  |  | GA/GG | 26 | 64 | 34 | 0.961 |
|  |  | Recessive | AA/GA | 56 | 133 | 71 |  |
|  |  |  | GG | 4 | 12 | 3 | 0.574 |
|  |  | Allele | A | 90 | 214 | 111 |  |
|  |  |  | G | 30 | 76 | 37 | 0.941 |
| **SOD2** | rs5746136 | Co-dominant | CC | 21 | 51 | 18 |  |
|  |  |  | TC | 27 | 66 | 36 |  |
|  |  |  | TT | 12 | 28 | 20 | 0.477 |
|  |  | Dominant | CC | 21 | 51 | 18 |  |
|  |  |  | TC/TT | 39 | 94 | 56 | 0.242 |
|  |  | Recessive | CC/TC | 48 | 117 | 54 |  |
|  |  |  | TT | 12 | 28 | 20 | 0.409 |
|  |  | Allele | C | 69 | 168 | 72 |  |
|  |  |  | T | 51 | 122 | 76 | 0.164 |
|  | rs8031 | Co-dominant | AA | 40 | 107 | 58 |  |
|  |  |  | AT | 18 | 37 | 15 |  |
|  |  |  | TT | 2 | 1 | 1 | 0.342 |
|  |  | Dominant | AA | 40 | 107 | 58 |  |
|  |  |  | AT/TT | 20 | 38 | 16 | 0.306 |
|  |  | Recessive | AA/AT | 58 | 144 | 73 |  |
|  |  |  | TT | 2 | 1 | 1 | 0.333 |
|  |  | Allele | A | 98 | 251 | 131 |  |
|  |  |  | T | 22 | 39 | 17 | 0.276 |

**Supplementary** **table 2. Polymorphisms associated with sensitivity to acute myeloid leukaemia** **[relapse](https://dict.youdao.com/search?q=relapse%0D%0A&keyfrom=fanyi.smartResult)**

| Gene | SNPs | Model | Genotype/  allele | No [relapse](https://dict.youdao.com/search?q=relapse%0D%0A&keyfrom=fanyi.smartResult) | [relapse](https://dict.youdao.com/search?q=relapse%0D%0A&keyfrom=fanyi.smartResult) | chi-square test  P-value |
| --- | --- | --- | --- | --- | --- | --- |
| **BCL2L11** | rs724710 | Co-dominant | CC | 71 | 36 |  |
|  |  |  | CT | 17 | 4 |  |
|  |  |  | TT | 1 | 0 | 0.456 |
|  |  | Dominant | CC | 71 | 36 |  |
|  |  |  | CT/TT | 18 | 4 | 0.153 |
|  |  | Recessive | CC/CT | 88 | 40 |  |
|  |  |  | TT | 1 | 0 | 1.000 |
|  |  | Allele | C | 159 | 76 |  |
|  |  |  | T | 19 | 4 | 0.139 |
|  | rs3789068 | Co-dominant | AA | 28 | 6 |  |
|  |  |  | GA | 43 | 23 |  |
|  |  |  | GG | 18 | 11 | 0.144 |
|  |  | Dominant | AA | 28 | 6 |  |
|  |  |  | GA/GG | 61 | 34 | 0.05 |
|  |  | Recessive | AA/GA | 71 | 29 |  |
|  |  |  | GG | 18 | 11 | 0.360 |
|  |  | Allele | A | 99 | 35 |  |
|  |  |  | G | 79 | 45 | 0.078 |
| **TP63** | rs4488809 | Co-dominant | TT | 26 | 13 |  |
|  |  |  | CT | 34 | 18 |  |
|  |  |  | CC | 29 | 9 | 0.532 |
|  |  | Dominant | TT | 26 | 13 |  |
|  |  |  | CT/CC | 63 | 27 | 0.707 |
|  |  | Recessive | TT/CT | 60 | 31 |  |
|  |  |  | CC | 29 | 9 | 0.245 |
|  |  | Allele | T | 86 | 44 |  |
|  |  |  | C | 92 | 36 | 0.321 |
|  | rs10937405 | Co-dominant | CC | 49 | 20 |  |
|  |  |  | TC | 30 | 16 |  |
|  |  |  | TT | 10 | 4 | 0.791 |
|  |  | Dominant | CC | 49 | 20 |  |
|  |  |  | TC/TT | 40 | 20 | 0.594 |
|  |  | Recessive | CC/TC | 79 | 36 |  |
|  |  |  | TT | 10 | 4 | 1.000 |
|  |  | Allele | C | 128 | 56 |  |
|  |  |  | T | 50 | 24 | 0.754 |
| **TP53BP2** | rs10916264 | Co-dominant | AA | 45 | 26 |  |
|  |  |  | GA | 37 | 10 |  |
|  |  |  | GG | 7 | 4 | 0.192 |
|  |  | Dominant | AA | 45 | 26 |  |
|  |  |  | GA/GG | 44 | 14 | 0.127 |
|  |  | Recessive | AA/GA | 82 | 36 |  |
|  |  |  | GG | 7 | 4 | 0.952 |
|  |  | Allele | A | 127 | 62 |  |
|  |  |  | G | 51 | 18 | 0.302 |
|  | rs798755 | Co-dominant | GG | 75 | 32 |  |
|  |  |  | GA | 12 | 5 |  |
|  |  |  | AA | 2 | 3 | 0.399 |
|  |  | Dominant | GG | 75 | 32 |  |
|  |  |  | GA/AA | 14 | 8 | 0.551 |
|  |  | Recessive | GG/GA | 87 | 37 |  |
|  |  |  | AA | 2 | 3 | 0.349 |
|  |  | Allele | G | 162 | 69 |  |
|  |  |  | A | 16 | 11 | 0.248 |
|  | rs1538140 | Co-dominant | CC | 28 | 14 |  |
|  |  |  | TC | 48 | 22 |  |
|  |  |  | TT | 13 | 4 | 0.803 |
|  |  | Dominant | CC | 28 | 14 |  |
|  |  |  | TC/TT | 61 | 26 | 0.692 |
|  |  | Recessive | CC/TC | 76 | 36 |  |
|  |  |  | TT | 13 | 4 | 0.474 |
|  |  | Allele | C | 104 | 50 |  |
|  |  |  | T | 74 | 30 | 0.537 |
| **PLAUR** | rs4251864 | Co-dominant | AA | 52 | 26 |  |
|  |  |  | GA | 32 | 11 |  |
|  |  |  | GG | 5 | 3 | 0.668 |
|  |  | Dominant | AA | 52 | 26 |  |
|  |  |  | GA/GG | 37 | 14 | 0.480 |
|  |  | Recessive | AA/GA | 84 | 37 |  |
|  |  |  | GG | 5 | 3 | 0.988 |
|  |  | Allele | A | 136 | 63 |  |
|  |  |  | G | 42 | 17 | 0.678 |
| **SOD2** | rs5746136 | Co-dominant | CC | 27 | 12 |  |
|  |  |  | TC | 38 | 19 |  |
|  |  |  | TT | 24 | 9 | 0.832 |
|  |  | Dominant | CC | 27 | 12 |  |
|  |  |  | TC/TT | 62 | 28 | 0.969 |
|  |  | Recessive | CC/TC | 65 | 31 |  |
|  |  |  | TT | 24 | 9 | 0.591 |
|  |  | Allele | C | 92 | 43 |  |
|  |  |  | T | 86 | 37 | 0.759 |
|  | rs8031 | Co-dominant | AA | 67 | 27 |  |
|  |  |  | AT | 22 | 13 |  |
|  |  |  | TT | 0 | 0 | 0.395 |
|  |  | Dominant | AA | 67 | 27 |  |
|  |  |  | AT/TT | 22 | 13 | 0.358 |
|  |  | Recessive | AA/AT | 89 | 40 |  |
|  |  |  | TT | 0 | 0 |  |
|  |  | Allele | A | 156 | 67 |  |
|  |  |  | T | 22 | 13 | 0.517 |
